# Metavert synergises with standard cytotoxics in human PDAC organoids and is associated with transcriptomic signatures of therapeutic response

**DOI:** 10.1016/j.tranon.2024.102109

**Published:** 2024-08-31

**Authors:** Jingyu An, Roma Kurilov, Teresa Peccerella, Frank Bergmann, Mouad Edderkaoui, Adrian Lim, Xu Zhou, Katrin Pfütze, Angela Schulz, Stephan Wolf, Kai Hu, Christoph Springfeld, Sadaf S. Mughal, Lenart Zezlina, Franco Fortunato, Georg Beyer, Julia Mayerle, Susanne Roth, Johannes Hulkkonen, Daniela Merz, Shigenori Ei, Arianeb Mehrabi, Martin Loos, Mohammed Al-Saeedi, Christoph W. Michalski, Markus W. Büchler, Thilo Hackert, Benedikt Brors, Stephen J. Pandol, Peter Bailey, John P. Neoptolemos

**Affiliations:** a Heidelberg University Hospital, Department of General, Visceral and Transplantation Surgery, Im Neuenheimer Feld 420, Heidelberg 69120, Germany; b Division of Applied Bioinformatics, German Cancer Research Center (DKFZ), Berliner Str. 41, Heidelberg 69120, Germany; c Institute of Pathology, Heidelberg University Hospital, Heidelberg, Germany; d Department of Medicine, Cedars-Sinai Medical Center and University of California at Los Angeles, Thalians W204 8700 Beverly Blvd. Los Angeles, California CA 90048, United States; e Sample Processing Laboratory, German Cancer Research Center (DKFZ) and National Center for Tumor Diseases (NCT) Heidelberg, Heidelberg, Germany; f NGS Core Facility, The German Cancer Research Center (DKFZ), Heidelberg, Germany; g Department of Medical Oncology, National Center for Tumor Diseases, University Clinic Heidelberg, Heidelberg 69120, Germany; h Department of Internal Medicine II, Ludwig-Maximilians-University of Munich, Germany; i Department of Gastroenterological Surgery, Tokai University School of Medicine, Kanagawa, Japan; j Botton-Champalimaud Pancreatic Cancer Centre, Lisbon, Portugal; k Department of General, Visceral and Thoracic Surgery, University Hospital Hamburg-Eppendorf, Hamburg, Germany; l German Cancer Consortium (DKTK), Core Center Heidelberg, Im Neuenheimer Feld 280, Heidelberg 69120, Germany; m Medical Faculty and Faculty of Biosciences, Heidelberg University, Im Neuenheimer Feld 234, Heidelberg 69120, Germany; n National Center for Tumor Diseases (NCT), Im Neuenheimer Feld 460, Heidelberg 69120, Germany

**Keywords:** Pancreatic cancer, Apoptosis, Autophagy, Molecular subtypes, GSK3-β, Histone deacetylases

## Abstract

**Background:**

Despite some recent advances, pancreatic ductal adenocarcinoma (PDAC) remains a growing oncological challenge. New drugs capable of targeting more than one oncogenic pathway may be one way to improve patient outcomes. This study characterizes the effectiveness of Metavert a first-in-class dual inhibitor of GSK3-β and histone deacetylase in treating PDAC as a single agent or in combination with standard cytotoxics.

**Methods:**

Thirty-six Patient-Derived Organoids (hPDOs) characterised by RNASeq and whole exome sequencing were treated with Metavert alone or in combination with standard cytotoxics. Transcriptomic signatures (TS) representing sensitivity to Metavert alone or sensitivity to Metavert + irinotecan (IR) were evaluated in 47 patient samples, chemo-naïve in 26 and post-chemotherapy in 21 (gemcitabine=5; FOLFIRINOX=14, both=2) with companion multiplexed immunofluorescence and RNASeq data.

**Results:**

Metavert combined with gemcitabine, irinotecan, 5FU, oxaliplatin, and paclitaxel was synergistic in the hPDOs. Basal-subtype hPDOs were more sensitive to Metavert alone whereas the Metavert+IR combination exhibited synergy in Classical-subtype hPDOs with increased apoptosis and autophagy. hPDO-derived TS evaluated in PDAC tissues demonstrated that Metavert-TS^Hi^ samples were enriched for mRNA splicing and DNA repair processes; they were associated with Basal-like tissues but also with GATA6^+ve^-chemo-naïve samples and were higher following gemcitabine but not FOLFIRINOX treatment. In contrast, Metavert+IR-TS^HI^ samples were enriched for TP53 pathways; they were associated with Classical-like pretreatment samples and with GATA6^+ve^/KRT17^+ve^ hybrid cell types following FOLFIRINOX, but not gemcitabine treatment, and were unrelated to transcriptional subtypes.

**Conclusions:**

Metavert as a single agent and in combination with irinotecan offers novel strategies for treating pancreatic cancer.

## Highlights

• Metavert is a first-in-class dual inhibitor of GSK3-β and histone deacetylase which has been shown to be an effective therapy for pancreatic cancer in preclinical studies.
• In planning for early phase clinical trials, we have explored the clinical potential for transcriptional signatures (TS) for Metavert monotherapy and in combination with standard cytotoxics.
• Metavert was found to synergise with irinotecan and to target Classical-like transcriptional subtype Patient-Derived Organoids (hPDOs) exhibiting increased apoptosis and autophagy.
• hPDO-derived transcriptional signatures of chemosensitivity to Metavert or Metavert plus irinotecan (IR) identified DNA repair and TP53 transcriptional programs as common pathways associated with Metavert or Metavert plus irinotecan sensitivity.
• hPDO-derived transcriptional signatures evaluated in chemo-naïve and post-chemotherapy PDAC tissues demonstrated that Metavert+IR-TS^HI^ samples were associated with Classical-like pretreatment samples and with GATA6^+ve^/KRT17^+ve^ hybrid cell types following FOLFIRINOX, but not gemcitabine treatment.

## Introduction

Pancreatic ductal adenocarcinoma (PDAC) is an aggressive cancer, with an overall five-year survival rate for all stages of only 12 % [1]. There is however improved survival when surgical resection can be undertaken and combined with systemic chemotherapy in the adjuvant and neoadjuvant settings [[2], [3], [4], [5], [6], [7]]. The most effective systemic therapies for metastatic and locally advanced PDAC are cytotoxics, comprising gemcitabine based therapies such as gemcitabine and nab-paclitaxel, or oxaliplatin based therapies such folinic acid, 5-fluorouracil (5-FU), irinotecan and oxaliplatin (FOLFIRINOX) [[8], [9], [10], [11], [12]]. Single targeted inhibitor therapy even when combined with chemotherapy has proved to be challenging due to complex redundant signaling [13,14]. Targeting more than one key signaling pathway may be an option provided toxicity is acceptable. Salvador-Barbero et al. showed that the CDK4/6 inhibitor palbociclib prevented cell-cycle re-entry after (but not before) taxol treatment, and when combined with the PARP inhibitor olaparib prevented tumor cell proliferation in two different PDX-derived cell lines [15].

A relatively novel approach is the rational design of single drugs capable of inhibiting more than one key oncogenic function. Metavert is a dual inhibitor that was developed to inhibit GSK3-β driven tumor-promotion via NF-κB activation, as well as blocking histone deacetylase (HDAC) classes to interfere with epithelial to mesenchymal transition (EMT), which otherwise would be enhanced by GSK3-β inhibition [[16], [17], [18], [19], [20], [21], [22], [23]]. The development of Metavert has so far been undertaken on two-dimensional cell lines and the KPC genetically engineered mouse model [16]. Herein we describe further investigations of Metavert using human PDAC derived three dimensional organoids (hPDO) that successfully model the genetic, morphological and biological properties of human tumor tissues [24,25]. We characterized a library of 36 in molecular and functional terms to develop an experimentally tractable preclinical model system for further investigating the mechanism of action of Metavert as a dual inhibitor. Furthermore, we developed de novo TS modeling drug response to Metavert (IC50) and Metavert plus irinotecan, respectively. Single Sample Gene Set Enrichment Analysis was performed to generate continuous TS scores, and further analyzed against a separate cohort of primary PDAC tissues characterized by RNASeq and multiplex immunofluorescence. The interactions between Metavert and currently used chemotherapeutics were shown to synergize with all the cytotoxics to a varying degree, and were associated with molecular subtypes, and specific Metavert and Metavert+irinotecan transcriptomic signatures. These findings open up new strategies for the treatment of pancreatic cancer.

## Methods and materials

**Patient Characteristics.** Thirty hPDOs were obtained from patients undergoing surgical resection plus three metastatic biopsy specimens from the University Clinic Heidelberg and three primary tumor biopsies from the Ludwig-Maximillian University (LMU), Munich. There were 29 hPDOs derived from patients that had not received any chemotherapy (chemo-naïve) and seven hPDOs generated from patients that had received prior chemotherapy. Companion RNASeq data was obtained from 35 of these - PDO h32 did not have companion RNASeq data. For testing TS derived from these hPDOs a separate cohort of 47 cryo-preserved PDAC tissues with both RNASeq and companion multiplex IF data previously described was used [26]. Samples from patients who received chemoradiation at any time were excluded from the present analysis. All samples were confirmed as PDAC tumors by specialist pancreatic cancer pathologists. Patient characteristics were extracted from the clinical database and anonymized. The patient demographics are provided in Table 1.

**Table 1:** Patient demographics and pathologic variables.

	Derived Organoids *N* = 36		Primary Tissue *n* = 47	
	Post-treatment group *N* = 7	Chemo-naïve group *N* = 29	Post-treatment group *N* = 21	Chemo-naïve group *N* = 26
PurIST transcriptomic subtype				
Classical	5	22	6	21
Basal	2	7	15	5
Gender ratio (m/f/na.)	4/3/0 (57/43/0)	13/16/0 (44.8/55.2/0)	11/10/0 (52.4/47.6/0)	14/12/0 (53.8/46.2/0)
Age in years*	66.0 (58.0 – 71.0)	73.5 (63.8 – 78.0)	63.0 (55.0 – 72.5.0)	64.5 (58.8.0 – 73.0)
CA 19–9 (U/mL) *	268.3 (16.4 – 1566)	183.4 (62.7 –527.9)	86.6 (6.9 – 451.0)	190.8 (75.0 – 665.7)
CEA (µg/L) *	2.5 (1.3 – 4.2)	16.3 (8.0 – 84.0)	2.2 (1.2 – 6.6)	2.5 (1.3 – 3.8)
Tissue resource				
Pprimary pancreatic tumor	6 (85.7)	27 (93.1)	21 (100)	26 (100)
Liver metastatic	1 (14.3)	2 (6.9)	0 (0)	0 (0)
Type of operation				
Pancreatoduodenectomy	3 (42.9)	13 (44.8)	9 (42.9)	13 (50)
Left pancreatectomy	1 (14.3)	8 (27.6)	3 (14.3)	6 (23.1)
Total pancreatectomy	1 (14.3)	2 (6.9)	9 (42.9)	7 (26.9)
NA	2 (28.6)	6 (27.6)	0 (0)	0 (0)
Tumor size				
≤2 cm (8th T1)	0 (0)	0 (0)	2 (9.5)	2 (7.7)
>2-≤4 cm (8th T2)	4 (57.1)	16 (55.2)	7 (33.3)	24 (92.3)
>4 cm (8th T3)	3 (42.9)	11 (37.9)	10(47.6)	0 (0)
NA	0 (0)	2 (6.9)	2 (9.5)	0 (0)
Lymph node status				
0 (8th N0)	3 (42.9)	7 (24.1)	7(33.3)	5 (19.2)
1–3 (8th N1)	1 (14.3)	11 (37.9)	7(33.3)	17 (65.4)
≥4 (8th N2)	3 (42.9)	9 (31.0)	7(33.3)	4 (15.4)
NA	0 (0)	2 (6.9)	0 (0)	0 (0)
M status				
M0	5 (71.4)	24 (82.8)	20 (95.2)	25 (96.2)
M1	2 (28.6)	5 (17.2)	1 (4.8)	1 (3.8)
NA	0 (0)	0 (0)	0 (0)	0 (0)
R margin status				
R0	3 (42.9)	23 (79.3)	4 (19.0)	8 (30.8)
R1	1 (14.3)	2 (6.9)	17(81.0)	18 (69.2)
Rx	0 (0.0)	1 (3.4)	0 (0)	0 (0)
NA	3 (42.9)	3 (10.3)	0 (0)	0 (0)
Chemotherapy before surgery				
FOLFIRINOX	3 (42.9)	NA	14(66.6)	NA
Gemcitabine-based	1 (14.3)	NA	5 (23.8)	NA
Combination	3 (42.9)	NA	2 (9.5)	NA

Values in parentheses are percentages; *values are median [IQR]; staging 8th AJCC edition.

**Biological agents.** Purchased reagents and antibodies are detailed in Supplementary Table 1.

**Cell culture experiments and organoid generation and propagation.** AsPC1, BxPC3, MiaPaCa2, and PANC1 cell lines were purchased from American Tissue Culture Collection (Manassas, VA) and grown in RPMI-1640 supplemented with 10 % fetal bovine serum and 1 % of antibiotic/antimycotics solution. The patient-derived Mayo-5289 cell line (PXC) (D Mukhopadhyay Department of Biochemistry and Molecular Biology, Mayo Clinic College of Medicine and Science, Jacksonville, FL 32224, USA) was cultured in Advanced DMEM/F-12 supplemented with 10 % fetal bovine serum (FBS), 1X l-glutaMAX, 10 mM HEPES. Cell lines were maintained in 75 cm^2^ flasks at 37 °C and 5 % CO2. The medium was changed twice a week, and cells were passaged when they achieved 80 % confluence. hPDOs were derived and cultivated according to Tuveson (https://tuvesonlab.labsites.cshl.edu/protocolsreagents/).

**RNA and DNA sequencing.** RNA and DNA was extracted from snap-frozen hPDO pellet samples using the AllPrep DNA/RNA/miRNA Universal Kit (Qiagen). Sanger sequencing. The organoid cell lines were initially checked for KRAS mutations by DNA Sanger sequencing. Primers sequences for amplification and sequencing of exons of the KRAS gene that contain the G12/13 codons were:

KRAS G12/13 Forward: 5′-CTGGTGGAGTATTTGATAGTG-3′

KRAS G12/13 Reverse: 5′-CTGTATCAAAGAATGGTCCTG-3′

PCR products were purified using a QIAquick PCR purification kit, then sent and sequenced by Eurofins and sequence analysis was undertaken using Mutation Surveyor software (SoftGenetics, USA).

RNA Seq. Sequencing libraries were prepared using the Illumina TruSeq mRNA stranded Kit following the manufacturer's instructions. Briefly, mRNA was purified from 500 ng of total RNA using oligo(dT) beads. Then poly(A)+ RNA was fragmented to 150 bp and converted to cDNA. The cDNA fragments were then end-repaired, adenylated on the 3′ end, adapter ligated and amplified with 15 cycles of PCR. The final libraries were validated using Qubit (Invitrogen) and Tapetstation (Agilent Technologies). 2 × 100 bp paired-end sequencing was performed on the Illumina NovaSeq 6000 according to the manufacturer's protocol. At least 54 Mio. reads per sample were generated.

Whole-Exome sequencing. Libraries were generated using the SureSelectXT Automation Reagent Kit and SureSelectXT Human All Exon v7 Capture Library (Agilent Technologies) following the manufacturer's instructions. In brief, 200 ng of gDNA was fragmented to ∼150 bp using a Covaris LE220 ultrasonicator (Covaris, Inc.). Subsequently, library preparation was performed on a Bravo automated liquid handler (Agilent Technologies) including end- repair, A- tailing, adaptor ligation and amplification. The concentration of amplified, adaptor- ligated DNA library was determined using the TapeStation (Agilent Technologies). In the subsequent steps 750 ng of amplified, adaptor- ligated DNA library was used for the hybridization reaction with the SureSelectXT All Exon v7 bait set. The DNA-library/bait hybrids were captured using streptavidin-coated magnetic beads (Dynabeads MyOne Streptavidin T1 by Thermo Fisher Scientific). Index tags were added in the course of PCR-amplification of the captured libraries.

**Pharmacological assay of cell lines and organoids.** The survival of cell lines was measured by MTT assay. The organoids were dissociated before plating 1000 cells in 10 μL Matrigel per well in white 96-well plates (Greiner). Cytotoxic drugs were dissolved in DMSO (concentrations were normalized to 0.25 % DMSO) and added 72 h after plating. All drugs were tested in triplicate at concentrations ranging from 1.0 × 10^−7^ to 1.0 × 10^−3^ mol/L for 5-FU (Sigma), irinotecan (Sigma) and oxaliplatin (Selleckchem); from 1.0 × 10^−10^ to 1.0 × 10^−6^ mol/L for gemcitabine (Sigma), paclitaxel (Selleckchem), and SN38 (Sigma) and for from 1.0 × 10^−7^ to 4.0 × 10^−4^ mol/L for Metavert (Royal Pharma, Mumbai, India). After 96 h of treatment, cell viability was assessed using the CellTiter-Glo 3D cell viability assay (Promega). A four-parameter log-logistic function with an upper limit equal to the mean of the DMSO values was fitted to the drug response curve and IC50’s were calculated.

**RNA Isolation and quantitative real-time RT-PCR.** RNA from cell lines and hPDOs was extracted by using the Trizol-Chloroform method. Two-step quantitative PCR (qPCR) was performed using a SYBR Green PCR Master Mix kit (Thermo Fisher). Expression values of the targeted gene in a given sample was normalized to the corresponding expression of GAPDH as ΔCT. The 2-ΔΔCt method was used to calculate relative expression of the targeted genes after the treatment. The primers for RT-qPCR reaction are listed in Supplementary Table 2.

**Histology for organoids.** Organoids were fixed in 4 % paraformaldehyde solution and embedded in paraffin. Sections were subjected to H&E and immunofluorescence (IF) staining. Images of H&E and IF staining were acquired using imaging system Tissue-FAXS software (Tissue Gnostics, Austria). H&E images were acquired using a 20X objective lens using a bright field. IF images were acquired using a 20X objective lens with light-emitting diodes (LED) and with specific light filters. IF images of negative control sections were used to set the appropriate gating to exclude background immunofluorescence and non-specific binding signals. The expression level of each protein was calculated by the percentage of protein-positive stained cells in DAPI-positive cells.

**Western blotting.** Protein extracts from organoids were lysed in RIPA Lysis Buffer 50 with a protease inhibitor cocktail (Sigma) and phosphatase inhibitor (Sigma) and quantified using Pierce BCA protein assay kit (ThermoFisher). Following SDS-PAGE and transfer to PVDF membranes (Bio-Rad, 1704273), the membranes were blocked in Tris-buffered saline containing 5 % BSA and 0.1 % Tween 20 (TBS-T) for 1 hour before incubation with the primary antibody overnight at 4 °C. After being washed three times in TBS-T and then incubated with species corresponding secondary antibodies (Anti-Mouse IgG, LI-COR, 1:10000; Anti-Rabbit IgG, LI-COR, 1:10000), the membrane was then visualized with an ODYSSEY CLx (LI-COR) image system.

**Statistical analyses**. GraphPad Prism was used to conduct statistical analyses utilizing the student *t-* test, one-way analysis of variance, and Fisher's exact test (GraphPad Software, La Jolla, CA). A P value less than or equal to 0.05 was considered statistically significant. The ggstatsplot R package was used to generate boxplots and assess the significance of sample comparisons. WES and RNAseq analysis: paired exome sequencing data was aligned, and SNVs and indels were called using the DKFZ-ODCF workflows.

Alignment and QC workflows: https://github.com/DKFZ-ODCF/AlignmentAndQCWorkflows

SNV calling workflow: https://github.com/DKFZ-ODCF/SNVCallingWorkflow

Indel calling workflow: https://github.com/DKFZ-ODCF/IndelCallingWorkflow

SNV and Indel output was converted from VCF to MAF format using a custom R script and then .maf files were summarized and visualized using maftools R package. CNV calling and CNV visualization was done using cnvkit tool. RNA-sequencing data were aligned and expression was quantified using the DKFZ-ODCF RNAseq workflow 28: https://github.com/DKFZ-ODCF/RNAseqWorkflow

For the downstream analysis we used log2(TPM+1) gene expression values. For classical/basal annotation we calculated PurIST score [27]; we assigned “classical” label for organoids with PurIST score ≤ 0.05, and “basal” label for organoids with PurIST score > 0.05. For expression visualization we used complexheatmap R package.

Synergistic scores: For each cell line and hPDOs tested in vitro drug synergistic assay, the synergistic score of drugs combination was calculated by SynergyFinder, a web application that uses essential functions of the R-package.

Signature Generation: Drug response data obtained from PDOs was used to generate gene expression signatures representing response to Metavert or synergistic response to Metavert plus irinotecan. To generate signatures we initially used the dNetPipeline function in the dnet R package and P-values representing the significance of differential gene expression between high (greater than 66 percentile) and low (lower than 33 percentile) response/synergy values to identify a maximum scoring subgraph from the STRING human functional protein association network. Each gene signature is the set of nodes (genes) representing the maximum scoring subgraph with coefficients of the P-values representing gene weights. Reactome pathway enrichment was assessed using the clusterProfiler R package.

Signature Scores: Gene signature scores representing the signed average of the set of genes making up each gene signature were calculated from normalized RNAseq data using the sig.score function as implemented in the genefu R package. Gene signature scores representing each patient sample were ordered by increasing value and signature genes visualized using the ComplexHeatmap R package.

## Results

**Genetic and transcriptomic profiling of organoids.** We established organoids from 36 patients with histologically confirmed PDAC in 31 cases from primary tumor following resection and in five cases from biopsies of the primary in two and three from liver metastases. (Table 1; Supplementary Figure 1). There were 29 hPDOs derived from patients that had not received any chemotherapy (chemo-naïve) and seven hPDOs generated from patients that had received prior chemotherapy (post-chemotherapy, h03, h20, h43, h44, h48, h51, h57). KRAS codon-12/13 mutations were found in 31 of 36 (86 %) hPDOs (Sanger sequencing in 34, whole exome sequencing (WES) in 29). Whole-exome sequencing in 29 organoids (7 post-chemotherapy and 22 chemo-naïve) revealed that the main driver mutated genes were KRAS (83 %), TP53 (66 %), CDKN2A (41 %), and SMAD4 (34 %), consistent with published studies (Supplementary Figure 1B) [12,24,28,29].WES was not possible in the other 7 organoids due to lack of blood samples for genomic comparison. Comparative genomic and transcriptomic profiling between complementary primary tissues and organoid samples is now shown in Supplementary Figures 2A and B. The mutational burden (single-nucleotide variants and insertion-deletion) was similar between the chemo-naïve and post-chemotherapy groups, although copy number variation, and copy number gain, was more apparent in the post-chemotherapy group indicating accumulated chromosomal instability (Supplementary Figures 1C and D). Transcriptome profiling identified nine PurIST Basal-like (h08, h69, h43, h63, h74, h03, h40, h36, and h33) and 27 Classical-like organoids (Supplementary Figure 1E) [27]. The morphology of all 36 organoids is shown in H&E images and brightfield images of a representative PDO (h33) in culture, immediately after generation from primary tissue at passage-0 and the other after passage-5, on culture days 1,3, 5, and 7 in Supplementary Figure 3.

**Phenotypic characterization of organoids.** Representative chemo-naïve hPDOs, eight Classical-like and one Basal-like (h03) were shown to express mRNA levels of HDACs 1–10 as well as GSK-3β (Supplementary Figure 4). Organoid h19 shown to be very chemo-resistant (see below, Fig. 2) had GSK-3β mRNA level 2–5 folds higher than in the other organoids tested. In addition, the mRNA levels of HDACs 5, 6, 7, 8, and 9 were the highest in the resistant organoid h19 compared to the other organoids. The increase in the level of HDAC9 was 5 to 50 folds compared to the other organoids. HDACs 1 to 4 and 10 were also highly expressed in the resistant h19 organoid. These data indicate a strong association between the levels of GSK-3β and HDACs, especially HDAC9, and the resistance to chemotherapy. Metavert induced a significant decrease in the mRNA level of HDAC9 (Supplementary Figure 4). Correspondingly with the reduced expression of HDAC9 there was an increase in the acetylated-H3K9 protein levels after Metavert treatment (Supplementary Figure 5). Previously mRNA levels of the stem cell markers Sox2, Nanog and CD133 were shown to be reduced in the MiaPaCa2, and BxPC3 cell lines by Metavert [16]. Here we showed reduced mRNA and protein levels in the cancer stemness markers Sox2 and CD44 in the organoids following Metavert treatment (Supplementary Figure 5).

**Fig. 1:**
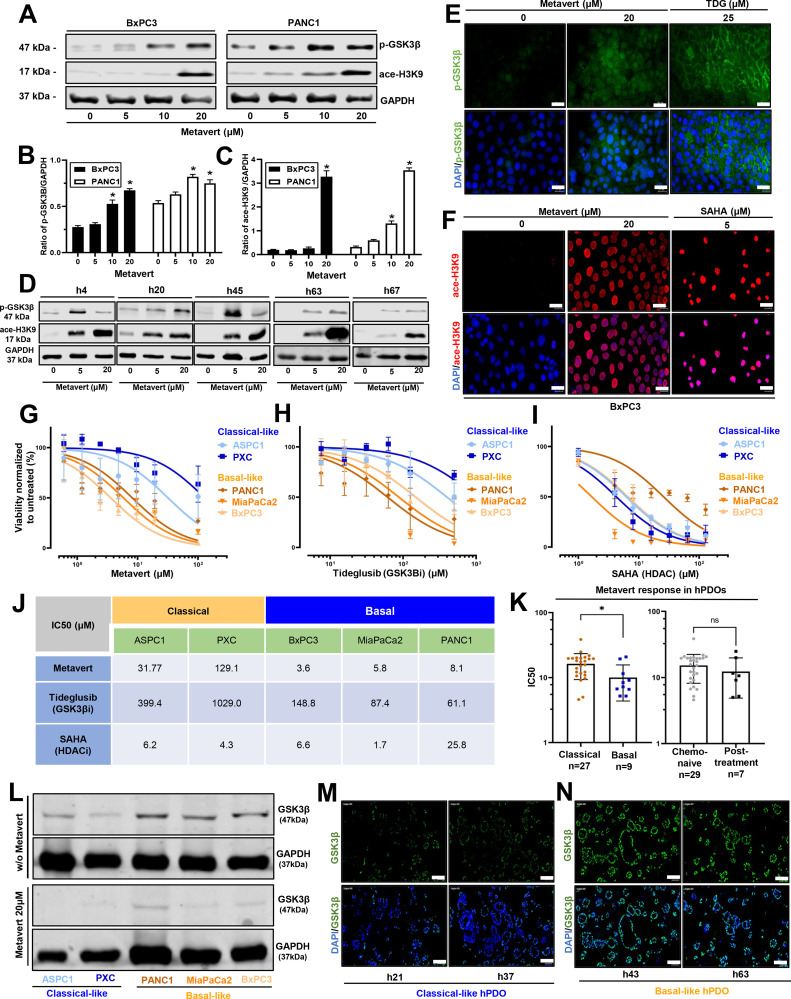
Cytotoxic effects of Metavert on Classical-like versus Basal-like selected cell lines and organoids. A-D: To confirm activity of Metavert protein levels of serine‑9 phosphorylation of GSK-3β (p-GSK3β) and acetylation of H3K9 (ace-H3K9) were measured in PDAC cell lines and hPDOs by western blotting after 72 h of treatment with the indicated concentrations of Metavert. Blots were re-probed for GAPDH to confirm equal loading (*n* = 3; *p < 0.05). E-F: To confirm intracellular activity of Metavert protein levels of p-GSK3β and ace-H3K9 were shown by immunofluorescence localization in the PDAC cell lines using the positive controls tideglusib (TDG) and SAHA, the respective inhibitors of GSK-3β and HDAC (scale bar=20 µm) stained for p-GSK-3β (green), ace-H3K9 (red), and DAPI (blue). G: MTT assays showed that the Base-like cell lines BxPC3, MiaPaCa2 and PANC1 were more sensitive to Metavert than the Classical-like AsPC1, PXC, cell lines. H-I: A similar MTT assay response in the cell lines to mono-inhibitor treatment for 72 h was found for the GSK-3β inhibitor tideglusib whereas all five cell lines were highly sensitive to the HDAC inhibitor SAHA (*n* = 3). J: The specific IC50 values in response to 72 h treatment with Metavert, tideglusib and SAHA in PDAC cell lines are shown (*n* = 3). K: Basal-like organoids were significantly more sensitive to Metavert compared to Classical-like organoids but there was no difference between the chemo-naive and post-treatment groups (mean ± SEM). L: Protein GSK-3β expression of PDAC cell lines re-probed for GAPDH shows increased expression in the Basal like cell-lines compared to the Classical-like cell lines. After 20 μM Metavert treatment for 72 h, there was a reduction of protein GSK-3β in both groups. (*n* = 3). (*p < 0.05, **p < 0.01, 2-sided unpaired t-test). M-N: Representative immunofluorescent co-localization images stained for GSK-3β (green) and DAPI (blue) also showing greater expression of GSK-3β in Basal-like compared to Classical-like organoids (scale bar=100 µm). (*p < 0.05, **p < 0.01, 2-sided unpaired t-test).

**Molecular subtype responses to Metavert.** First, we compared the effects of Metavert on the ASPIC1 and PXC Classical-like and the BxPC3, MiaPaCa2 and PANC1 Basal-like cells. The protein and mRNA expression levels of serine‑9 phosphorylation of GSK-3β (p-GSK3β) and acetylation of H3K9 (ace-H3K9) in BxPC3 and PANC1 cell lines were significantly upregulated after Metavert (Fig. 1A-C). Increased cellular protein expression of p-GSK-3β and ace-H3K9 in BxPC3 cells of Metavert was demonstrated by immunofluorescence and reproduced using tideglusib (TDG) and SAHA the respective inhibitors of GSK-3β and HDAC (classes I and II) (Fig. 1E and F). The Basal-like subtype cell lines were relatively more sensitive to Metavert than the Classical-like cell lines (Fig. 1G) [30,31]. The Basal-like cell lines were also more sensitive to GSK-3β inhibition by Tideglusib but not to HDAC acetylation by SAHA (Fig. 1H-J). Second, we showed that in organoids there was also increased protein levels of p-GSK3β inhibitory phosphorylation and ace-H3K9 after Metavert (Fig. 1D). As in the cell lines the Basal-like hPDOs were more sensitive to Metavert than Classical-like hPDOs (Fig. 1K). The increased sensitivity of Basal-like cells may be linked to intrinsically higher GSK-3β protein expression, with reduction after Metavert treatment. (Figs. 1l-N).

**Fig. 2:**
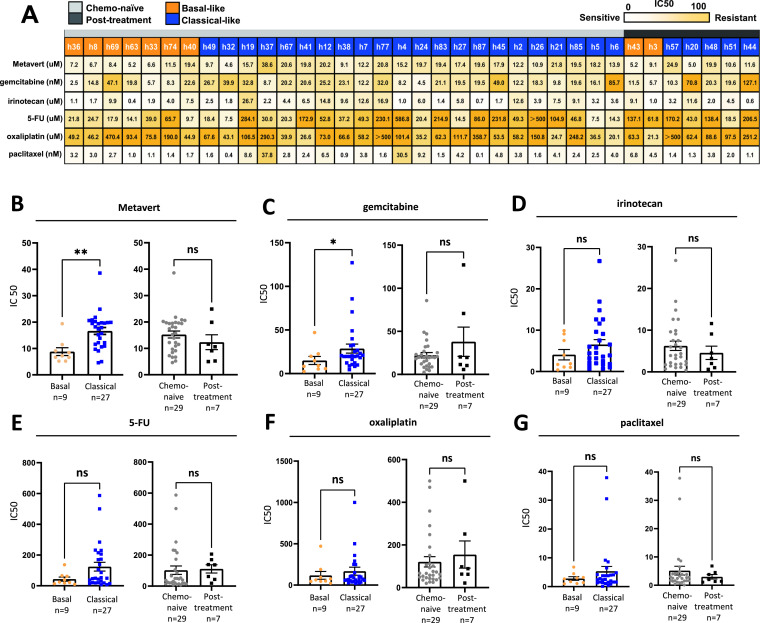
Metavert increases the sensitivity to chemotherapy. A: Different IC50 distribution for the 36 hPDOs treated with Metavert and five standard cytotoxics. The white to yellow scale represents the relative sensitive and resistant responses as a continuum. Each box shows the actual IC50 value (*n* = 3). B-G: Individual drug organoid sensitivities, comparing Basal versus Classical-like hPDOs and organoids derived from chemo-naive versus post-chemotherapy PDAC tissues. Metavert (B) (*p* = 0.001) and gemcitabine (C) (*p* = 0.046) were each more potent in the Basal-like organoids.

**Organoid sensitivity to Metavert and individual cytotoxics.** The therapeutic response to 5-FU, oxaliplatin, and irinotecan, gemcitabine and paclitaxel cytotoxic reagents was assessed in all 36 hPDOs. Drug response of organoids from different subtypes and treatment groups showed heterogeneity both for the same drug and the different drugs, consistent with previous studies (Fig. 2) [24,26,32]. Basal hPDOs were more sensitive to Metavert and gemcitabine.

**Metavert induces autophagy mediated apoptosis.** Metavert-treated organoids showed morphological apoptosis-like characteristic cell blebbing and shrinkage, nuclear fragmentation, condensation and fragmentation of genetic materials (6 A). In BxPC3 and PANC1 cell lines Metavert demonstrated features of apoptosis with cleavage of poly-ADP-ribose polymerase (c-PARP), and autophagy with lipidation of microtubule-associated protein 1 light chain 3 to generate the electrophoretically mobile form II (LC3-II), but not necroptosis as shown by the marker levels of phosphorated Mixed lineage kinase domain-like protein (p-MLKL) (Supplementary Figure 6 B-E), and supported by immunofluorescent levels of consisted of staining of c-PARP, cleaved-Caspase3, and p-MLKL with corresponding death inducers as positive control (Supplementary Figure 6 F-H) [33,34].

As autophagy is a dynamic multistep process and elevated LC3-II levels are linked to autophagosome production or turnover we next evaluated Metavert-induced autophagic flux using a tandem mCherry-GFP-LC3 reporter fluorescence experiment (Supplementary Figure 6 I) [35]. Fluorescent microscopy revealed mCherry-GFP-LC3 as a diffuse cytoplasmic pool for the untreated group. The homogeneous fusion of the red and green fluorescence was exhibited. The treated group showed that Metavert exposure dose-dependently led to significantly increased numbers of mCherry-tagged LC3 protein puncta. In contrast, the number of GFP puncta did not increase due to the acid environment resulting from autophagosome fusion and lysosome. This observation suggests that Metavert stimulates the formation of the autophagosomes and activates the autophagic flux in BxPC3 cells. In contrast, additional yellow labeled puncta (overlay between mCherry and GFP puncta) were identified in the negative control group due to the fusion block of the autophagosome and lysosome by chloroquine. In addition, after blocking the fusion of autophagosome and lysosome pretreated with chloroquine, Metavert treatment still caused upregulated LC3-II expression in a dose-dependent fashion. Supplementary Figure 6 J shows an increase in protein levels of both LC3-I and LC3-II with increasing does of Metavert when degradation is blocked by adding chloroquine. We next performed a rescue assay on BxPC3 and PANC1 cell lines by pretreatment with necroptosis inhibitors (necrostatin-1), an apoptosis inhibitor (ZVAD-FMK) plus autophagy inhibitors chloroquine and 3-methyladenine before receiving Metavert treatment. Only the early-stage autophagy inhibitor 3-methyladenine restored cell viability (Supplementary Figure 6 K).

**Fig. 3:**
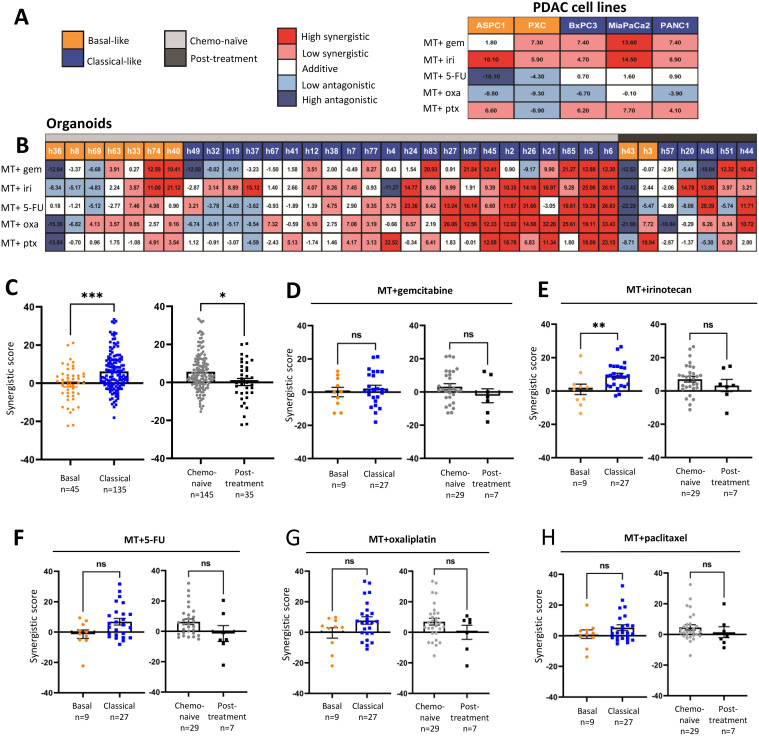
Synergistic effects of Metavert with cytotoxics in cell lines and organoids.

**Synergistic effects of Metavert and cytotoxics.** The dose-dependent matrix viability assay in PDAC cell lines was initially used to determine drug sensitivity at various dose combinations to assess any potential synergy effect between Metavert and the cytotoxic drugs. Five established cell lines received Metavert in combination with either gemcitabine, irinotecan, 5-FU, oxaliplatin, or paclitaxel in different doses (Fig. 3). Overall Metavert exhibited a relatively strong but variable synergistic effect when combined with gemcitabine, irinotecan and paclitaxel in all five of the PDAC cell lines. A variable synergistic effect of Metavert when combined individually with all five tested cytotoxics was also seen across the 36 hPDOs. The synergistic effect was greater in the Classical than the Basal-like organoids and similarly in the chemo-naïve than in the post-treatment derived organoids. Synergy was observed in 22 of the 36 hPDOs treated with gemcitabine, in 24 with irinotecan, 18 with 5-FU, 22 with oxaliplatin, and in 15 treated with paclitaxel. The synergistic effect of Metavert with irinotecan, was also most evident in the Classical-like organoids. Metavert greatly increased apoptosis and autophagy when combined with irinotecan (Supplementary Figure 7). There was no difference in IC50 values and synergistic between organoids derived from primary tumors and those derived from metastases (Supplementary Figure 8A). Also, there were no significant differences in the synergy scores in combining Metavert with individual cytotoxics between those organoids dichotomized as Metavert relatively sensitive and relatively non-sensitive. (Supplementary Figure 8B).

The synergistic score is calculated by the Zero interaction potency (ZIP) model. Red indicates a high synergistic effect, white a neutral effect is white, and dark blue a high antagonistic effect. **A:** the Basal-like AsPC1, and PXC cells, and the Classical-like BxPC3, MIA PaCa2, and PANC1 cells, were treated for 72 h with up to 19.2 μM cytotoxic drug (*n* = 3). The strongest synergy across all cell lines was the combination of Metavert+IR. **B:** The synergistic scores of Metavert combined with other drugs (*n* = 3). Values over 10 indicating strong synergy are depicted in dark red, and values over three but less than ten (indicating low synergy are depicted in light red. Scores between −3 and 3 were considered as dual drug additives. Values <10 indicating strong antagonistic are depicted in dark blue, and values less than −3 but more than −10 indicating low synergy are depicted in light blue. **C:** Classical-like organoids were more synergistic than Basal-like hPDOs (*p* = 0.001), and organoids derived from chemo-naive tissues were also more synergistic than organoids derived from tissues following chemotherapy (*p* = 0.017). **d-H:** Synergistic scores for individual cytotoxic parings with Metavert. **E:** Metavert+IR had significantly had significantly higher scores in the Classical-like hPDOs (*p* = 0.0095).

**Fig. 4:**
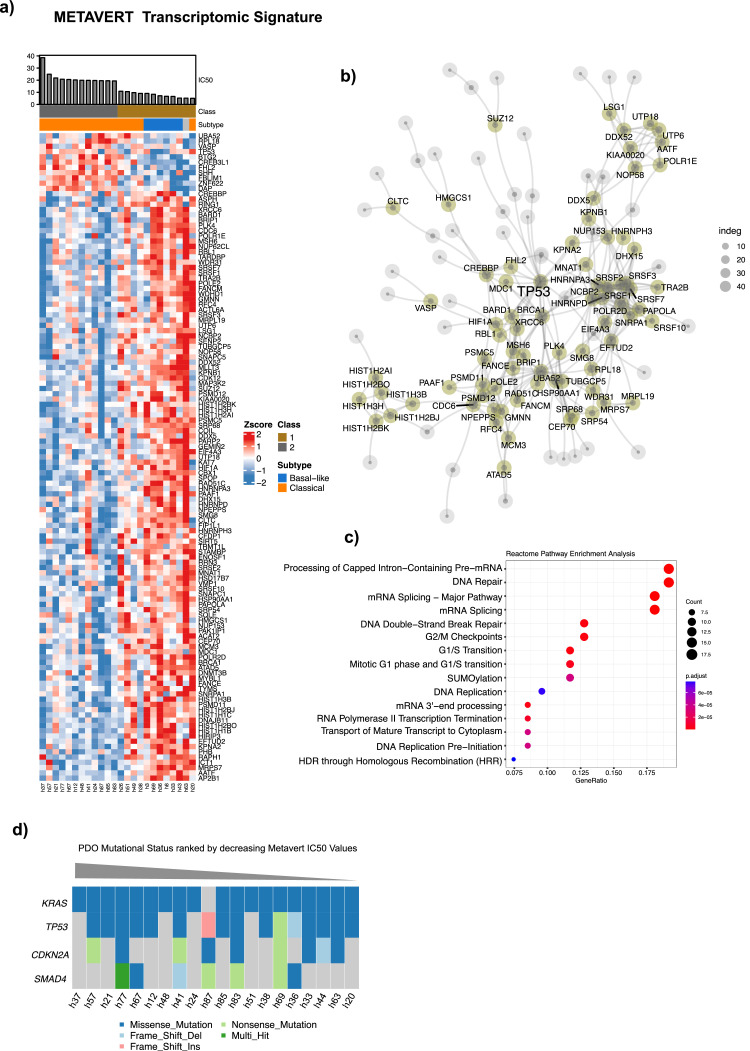
Metavert transcriptomic signature and network analysis.

**Fig. 5:**
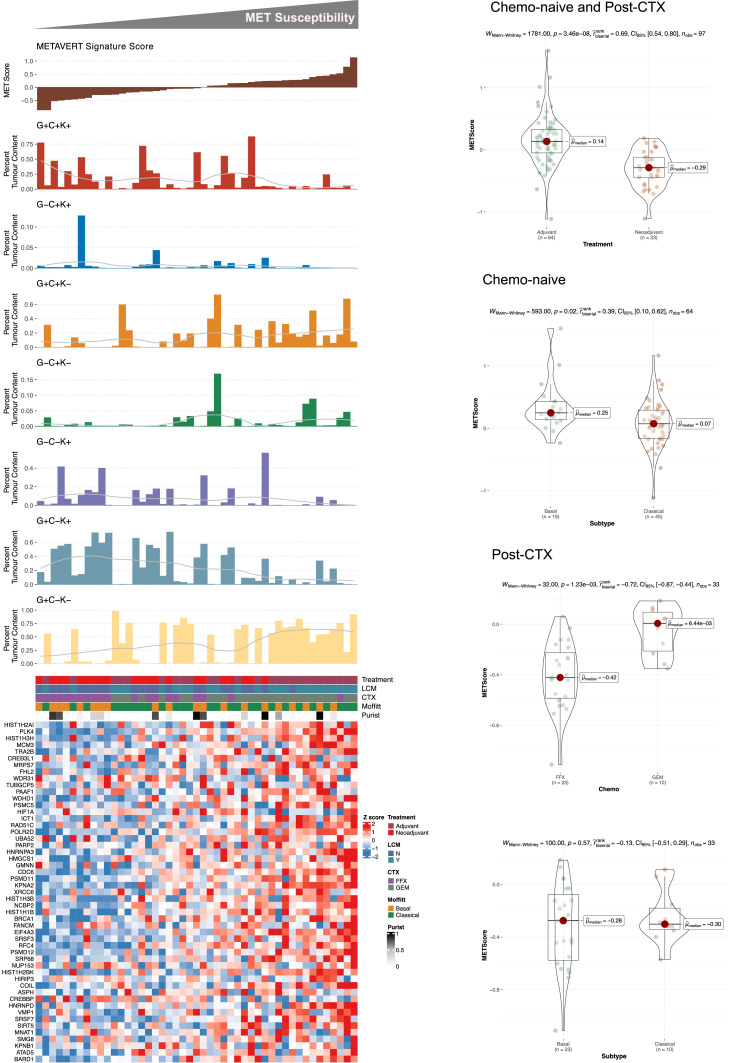
Metavert transcriptomic signature associations in organoid transcriptomic subtypes and treatment groups.

**Fig. 6:**
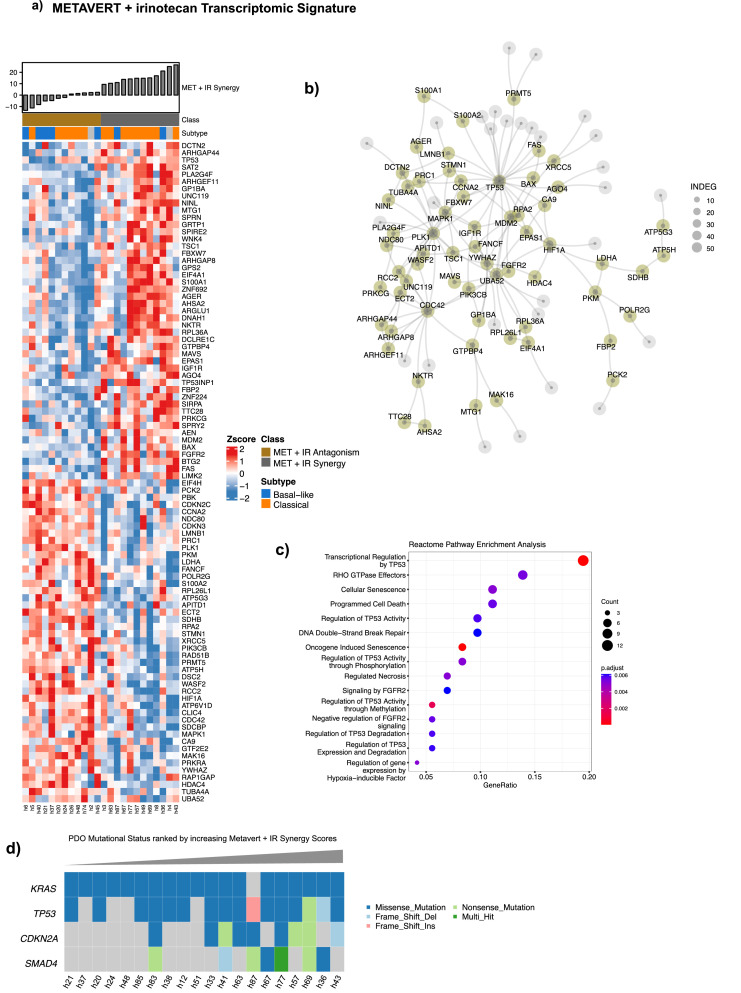
Metavert+irinotecan signature and network analysis.

**Fig. 7:**
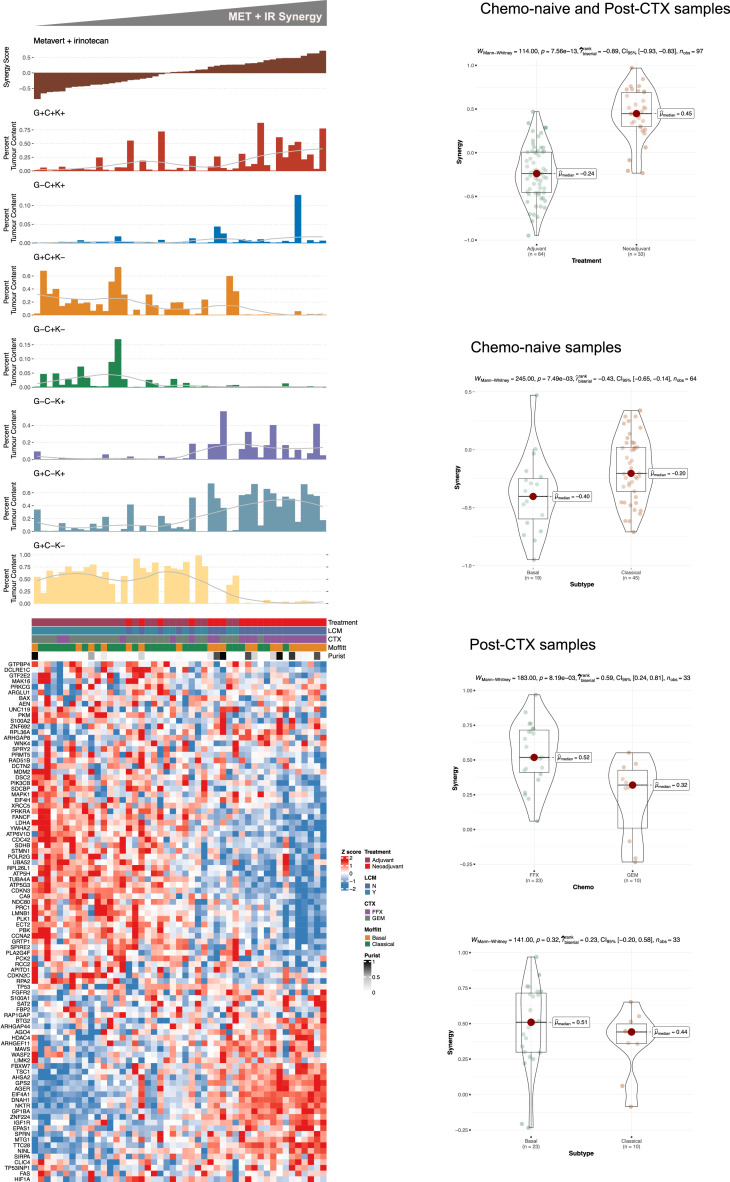
Metavert+irinotecan transcriptomic signature associations in organoid transcriptomic subtypes and treatment groups.

**Transcriptomic signatures.** Drug response data obtained from hPDOs (IC50s) were used to generate continuous TS - by Single Sample Gene Set Enrichment Analysis - representing response to Metavert or synergistic response to Metavert+IR. These TSs were evaluated in 47 primary PDAC tissues that had both RNASeq analysis and multiplex immunofluorescence (IF) including GATA6, CYP3A5 and KRT17 and previously reported by our laboratory [26]. The clinical data are summarised in Table 1. Metavert-TS^HI^ scores were significantly associated with hPDOs exhibiting a Basal-like phenotype and were enriched for mRNA splicing and DNA repair molecular processes (Fig. 4). Metavert-TS^HI^ scores were higher in Basal-like transcriptional states in resected chemo-naïve patient samples (Fig. 5). Metavert-TS^HI^ scores were higher following neoadjuvant chemotherapy with gemcitabine but not with FOLFIRINOX. In chemo-naïve samples whilst Metavert-TS^HI^ scores were associated with a Basal-like phenotype they were but also associated with GATA6^+ve^ tissues. Metavert-TS^HI^ scores were not significantly associated with transcriptional states in post-chemotherapy patient samples. In contrast, Metavert+IR-TS^HI^ scores were enriched for TP53 regulatory processes and or functions and were significantly higher in post-chemotherapy patient samples (Fig. 6). Metavert+IR-TS^HI^ scores were significantly associated with Classical-like transcriptional states in resected chemo-naïve patient samples, consistent with the findings in hPDOs (Fig. 7). Metavert+IR-TS^HI^ scores were significantly higher in post-chemotherapy patient samples after FOLFIRINOX but not after gemcitabine and were unrelated to transcriptional subtype. Metavert+IR-TS^HI^ scores were associated with GATA6^+ve^/KRT17^+ve^ hybrid cell types in predominantly post-chemotherapy patient samples.

a) Heatmap showing the relative expression of signature genes between Metavert (MET) High and Low IC50 values. High and Low MET IC50 groups represent the upper 33 % quantile of IC50 and lower 33 % quantile of Metavert IC50 values, respectively. b) Network of signature genes representing the maximum scoring subgraph obtained from the set of genes differentially expressed between high and low MET IC50 groups. Nodes (circles) represent genes and lines represent functional protein associations as curated by STRING. The degree of network centrality is shown by the size of each node. Node size denotes a higher degree of interconnectedness. c) Reactome pathway enrichment analysis showing significantly enriched pathways associated with the set of MET signature genes. P-values are adjusted by Bonferroni-Hochberg correction. d) Oncoprint plot showing most recurrent driver mutations in PDOs ranked by decreasing METAVERT IC50 value.

a) Top panel, Bar charts showing Metavert (MET) score and percent tumor enrichment of GATA6/CYP3A/KRT17 cell populations as determined by multiplexed IF. A LOESS regression line has been added to each tumor cell enrichment bar plot. Bottom panel, Heatmap showing the relative mRNA expression of MET signature genes. Heatmap annotation shows sample characteristics. Patient samples in top and bottom panels are identical (*n* = 47) and similarly ordered according to MET sensitivity. b) Boxplots showing MET signature scores stratified according to treatment as indicated. c) Boxplots showing MET signature scores stratified according to the Moffitt subtype in chemo-naive patient samples. d) Boxplots showing MET signature scores stratified according to treatment type in post-CTX patient samples. e) Boxplots showing MET signature scores stratified according to the Moffitt subtype in post-CTX patient samples. Mann-Whitney rank sum test (two-sided) P-values are shown on the plots. Boxplots show the median (line), the interquartile range (IQR) between the 25th and 75th percentiles (box) and 1.5 × the IQR ± the upper and lower quartiles. P-values were not adjusted for multiple testing. d) Oncoprint plot showing most recurrent driver mutations in PDOs ranked by increasing METAVERT + irinotecan synergy scores.

a) Heatmap showing the relative expression of signature genes between Metavert (MET) plus irinotecan High and Low synergy scores. High and Low synergy scores represent the upper 33 % quantile and lower 33 % quantile of synergy scores, respectively. b) Network of signature genes representing the maximum scoring subgraph obtained from the set of genes differentially expressed between samples exhibiting high and low MET plus irinotecan synergy scores. Nodes (circles) represent genes and lines represent functional protein associations as curated by STRING. The degree of network centrality is shown by the size of each node. Node size denotes the degree of interconnectedness. c) Reactome pathway enrichment analysis showing significantly enriched pathways associated with the set of MET plus irinotecan signature genes. P-values are adjusted by Bonferroni-Hochberg correction.

a) Top panel, Bar charts showing Metavert (MET) plus irinotecan synergy scores and the percent tumor enrichment of GATA6/CYP3A/KRT17 cell populations as determined by multiplexed IF. A LOESS regression line has been added to each tumor cell enrichment bar plot. Bottom panel, Heatmap showing the relative mRNA expression of MET plus irinotecan signature genes. Heatmap annotation shows sample characteristics. Patient samples in top and bottom panels are identical (*n* = 47) and similarly ordered according to MET plus irinotecan synergy score. b) Boxplots showing MET plus irinotecan synergy scores stratified according to treatment as indicated. c) Boxplots showing MET plus irinotecan synergy scores stratified according to the Moffitt subtype in chemo-naive patient samples. d) Boxplots showing MET plus irinotecan synergy scores stratified according to treatment type in post-CTX patient samples. e) Boxplots showing MET plus irinotecan synergy scores stratified according to the Moffitt subtype in post-CTX patient samples. Mann-Whitney rank sum test (two-sided) P-values are shown on the plots. Boxplots show the median (line), the interquartile range (IQR) between the 25th and 75th percentiles (box) and 1.5 × the IQR ± the upper and lower quartiles. P-values were not adjusted for multiple testing.

## Discussion

The most effective systemic therapy for PDAC is based on cytotoxic regimens but the ceiling in terms of survival has been reached with triplet therapies (NALIRIFOX and FOLFIRINOX) [5,9,11]. Targeted therapies based on specific genetic alterations such as BRCA1/2 mutations, microsatellite instability (MSI-H) or deficient mismatch repair, and NTRK1/2/3 fusions benefit no >5 % of all patients [[12], [13], [14]]. This study characterized the effectiveness of a first-in-class dual inhibitor named Metavert in treating pancreatic cancer. We confirmed strong inhibition of HDAC9, and increased GSK3-β serine‑9 phosphorylation and H3K9 acetylation in human derived PDAC organoids previously shown only in established cell lines and KPC mice^16^. Cell death was observed in all of the organoids in response to Metavert with autophagy mediated apoptosis being the principal mechanism, which was increased by the synergistic combination of Metavert with irinotecan. Both GSK3-β and HDAC inhibitors have been shown to target autophagy. Here we demonstrate that susceptibility to Metavert or Metavert plus irinotecan is associated with an increased enrichment of pathways associated with DNA damage repair, transcriptional regulation by TP53 and RNA splicing. TP53 mutations which occur in greater than 70 % on PDAC can lead to impaired regulation of autophagy, increased replications stress, aberrant ribosome biogenesis and genomic instability [12,28,29]. Metavert treatment of human organoids also strongly downregulated mRNA and protein levels of the cancer stem cell markers CD44 and SOX2 associated with EMT and chemotherapy resistance. A group of Basal-like subtype cell lines and hPDOs expressing relatively higher GSK-3β protein levels were more sensitive to Metavert than the Classical-subtype. This observation suggests that the PDAC molecular subtype as shown in cell-lines and hPDOs may influence their response to Metavert. This supports the discovery by Brunton et al. using patient derived cell lines that GSK-3β inhibition is positively correlated with their molecular subtype and subtype-specific GSK-3β protein expression [30]. It has become apparent that PDAC tumors undergo plasticity over time and in response to certain types of chemotherapy notably FOLFIRINOX shifting to more Basal-like subtype [12,26,36]. In which case the use of chemotherapy in Basal-like tumors could be particularly amenable to Metavert treatment.

The increased PDAC cell killing by GSK-3β inhibition and gemcitabine in cell lines has been ascribed to regulation of the TopBP1/ATR/Chk1 DNA damage response pathway [16,36]. In the present study using hPDOs we have shown synergistic cytotoxicity of Metavert with standard cytotoxics used to treat pancreatic cancer. The combination of Metavert and irinotecan displayed the most significant synergistic anti-tumor effect in Classical-subtypes, and greatly increased apoptosis and autophagy. Irinotecan is a prodrug that is converted into the active metabolite SN-38 and resistance appears to be tumor-cell intrinsic metabolism by uridine diphosphate glucuronosyltransferase 1A1 and cytochrome P450 mediation [37,38]. An insight into the synergistic action of Metavert with irinotecan is that GSK-3β inhibitors have been shown to activate the WNT/β-catenin pathway that regulate the expression of CYP2E [39].

The finding that Metavert-TS^HI^ scores were significantly associated with hPDOs exhibiting a Basal-like phenotype and were enriched for mRNA splicing and DNA repair molecular processes is consistent with our experimental and earlier published findings linking GSK3-β inhibition with Basal-like transcriptional states [30]. The association of Metavert-TS^HI^ and Metavert+IR-TS^HI^ scores with different molecular profiles may open up novel strategies for treating pancreatic cancer, as different cell regulatory mechanisms appear to be susceptible to single agent Metavert (mRNA splicing and DNA repair molecular processes) or to Metavert+IR (regulation of TP53 molecular processes). Notably Metavert+IR-TS^HI^ scores were significantly higher in post-chemotherapy patient samples after FOLFIRINOX but not after gemcitabine and were associated with GATA6^+ve^/KRT17^+ve^ hybrid cell types that have recently been identified as persister cells following with resistance to irinotecan therapy (and hence FOLFIRINOX) [26]. Precision clinical trials using Metavert alone or in combination with irinotecan are now being developed incorporating these signatures.

## Conclusions

Therapies targeting residual disease represent a major therapeutic opportunity for PDAC. Here we identify a novel drug combination with the potential to target previously characterized chemotherapy resistant persister cell populations. This study also provides robust transcriptional signatures for selecting patients for Metavert or Metavert plus irinotecan therapy, paving the way for biomarker driven clinical trials. Autophagy is a common feature of advanced PDAC and a promising therapeutic target with numerous ongoing clinical trials targeting the autophagy-lysosome pathway. Our work points to Metavert alone or in combination with irinotecan as an effective therapy targeting autophagic cells. These findings therefore define novel drug combinations and companion biomarkers for the treatment of PDAC.

## Ethics approval and consent to participate

The study has been approved by the Ethics Committee of Heidelberg University for use of pancreatic cancer tissue and organoid generation (Project Nos. S-018/2020, S-708/2019 and S-083/2021) and the Ludwig Maximilian University (LMU) of Munich. All patients provided informed consent for use of their tissue and clinical data in accordance with the Principles of Helsinki.

## Consent for publication

All authors have given their consent for publication.

## Availability of data and materials

Analytic methods, study materials and data are clearly described; any individual data are available on written request. Sequencing data is available at: https://ega-archive.org/studies/EGAS00001007143

## Funding

Grant Support: Grants have been received by SP, NCI - P01CA233452, ME, DoD- PA210175; FF, MWB, TH, and JPN, Heidelberger Stiftung Chirurgie and the German Ministry for Education and Research [BMBF]- 01GS08114 and 01ZX1305C; JPN and CS, BMBF-01KD2206J and Stiftung Deutsche Krebshilfe-70113834.

## Authors’ contributions

The study was conceived by JPN, SP, ME, MWB; lab work undertaken in HD by JA, TP, XZ, and SE, and in CSMC by ME, and AL; KP extracted the RNA and DNA; AS, and SW undertook the RNASeq and WES; FF provided advice regarding autophagy; pathology review by FB; oncology review by CS; clinical data review by KH, CM, and DM; clinical tissue provided by AM, ML, MA-S, MWB, TH, SR, and JH; initial support for organoids by GB and JM; bioinformatics by RK, BB, and PB; initial draft by JA, PB, and JPN, with subsequent input by all co-authors.

## CRediT authorship contribution statement

**Jingyu An:** Formal analysis, Data curation, Conceptualization. **Roma Kurilov:** Formal analysis. **Teresa Peccerella:** Formal analysis, Data curation. **Frank Bergmann:** Formal analysis. **Mouad Edderkaoui:** Formal analysis, Data curation, Conceptualization. **Adrian Lim:** Resources, Data curation. **Xu Zhou:** Formal analysis, Data curation. **Katrin Pfütze:** Data curation. **Angela Schulz:** Data curation. **Stephan Wolf:** Formal analysis, Data curation, Conceptualization. **Kai Hu:** Data curation. **Christoph Springfeld:** Data curation, Conceptualization. **Sadaf S. Mughal:** Formal analysis. **Lenart Zezlina:** Formal analysis. **Franco Fortunato:** Methodology, Data curation. **Georg Beyer:** Data curation. **Julia Mayerle:** Data curation. **Susanne Roth:** Data curation. **Johannes Hulkkonen:** Data curation. **Daniela Merz:** Data curation. **Shigenori Ei:** Data curation. **Arianeb Mehrabi:** Data curation. **Martin Loos:** Formal analysis, Data curation, Conceptualization. **Mohammed Al-Saeedi:** Data curation. **Christoph W. Michalski:** Data curation. **Markus W. Büchler:** Formal analysis, Data curation, Conceptualization. **Thilo Hackert:** Data curation. **Benedikt Brors:** Methodology, Data curation. **Stephen J. Pandol:** Formal analysis, Data curation, Conceptualization. **Peter Bailey:** Writing – review & editing, Supervision, Project administration, Formal analysis, Data curation. **John P. Neoptolemos:** Writing – original draft, Supervision, Formal analysis, Conceptualization.

## Declaration of competing interest

ME and SP are involved in clinical development of Metavert with Avenzoar Pharmaceuticals. The remaining authors declare that they have no competing interests.

## Appendix Supplementary materials

Image, application 1
