# Supplementary material for: Metavert synergises with standard cytotoxics in human PDAC organoids and is associated with transcriptomic signatures of therapeutic response
Source: Transl Oncol. 2024 Aug 31;49:102109. doi: 10.1016/j.tranon.2024.102109 (PMC11402625; doi:10.1016/j.tranon.2024.102109)
Supplement: Supplementary file 1 [file mmc1.docx]

# **SUPPLEMTARY MATERIAL**

**Title: Metavert synergises with standard cytotoxics in human PDAC organoids and is associated with transcriptomic signatures of therapeutic response**

**Jingyu An et al 2024**

**Contents**

**Supplementary Table 1. Antibodies for IF/IHC and WB.**

**Supplementary Table 2. PCR primer sequences.**

**Supplementary Figure 1. Clinical and molecular details of the 36 human derived pancreatic cancer derived organoids.**

**Supplementary Figure 2. Genomic and transcriptomic comparison between organoids and matched primary tissue.**

**Supplementary Figure 3. Morphology of organoids from H&E and brightfield images of PDOs.**

**Supplementary Figure 4. RNA expression levels of HDACs and GSK-3β, and HADC-9 levels following Metavert treatment in organoids.**

**Supplementary Figure 5. Western blots of CD44, SOX2, and ace-H3K9, and CD44 and SOX2 mRNA levels before and after Metavert treatment.**

**Supplementary Figure 6. Induction of autophagy and apoptosis contributes to the anticancer activity of Metavert.**

**Supplementary Figure 7. Metavert synergistically increased apoptosis and autophagy when combined with irinotecan.**

**Supplementary Figure 8. Synergistic comparison based on tissue resources and Metavert response.**

**Legends for Supplementary Figures 1-8**

**Supplementary Table 1. Antibodies for IF/IHC and WB**

***The list of primary antibodies for IF and IHC.***

| Antibodies | Vendor | Catalog No. | Dilution Ratio | Host |
| --- | --- | --- | --- | --- |
| KRT19 | Abcam | ab-7755 | 1:100 | Mouse |
| Ace-H3K9 | Cell Signaling | 9649 | 1:200 | Rabbit |
| p-GSK-3β(Ser9) | Cell Signaling | 5558 | 1:100 | Rabbit |
| PRELP | Invitrogen | PA5-78631 | 1:100 | Rabbit |
| p-MLKL | Abcam | Ab187091 | 1:100 | Rabbit |
| c-PARP | Cell Signaling | 5625 | 1:100 | Rabbit |
| c-Caspase3 | Abcam | Ab-2302 | 1:100 | Rabbit |

The primary antibodies for IF and IHC were diluted in antibody diluents (with Background Reducing Components). The primary antibodies for WB were diluted in 0.1% TBS-T with 5% BSA.

***The list of primary antibodies for WB.***

| Antibodies | Vendor | Catalog No. | Dilution Ratio | Host |
| --- | --- | --- | --- | --- |
| Ace-H3K9 | Cell Signaling | 9649 | 1:1000 | Rabbit |
| p-GSK3β(Ser9) | Cell Signaling | 5558 | 1:1000 | Rabbit |
| GSK-3β | Cell Signaling | 12456 | 1:1000 | Rabbit |
| WNT3A | Cell Signaling | 2391 | 1:1000 | Rabbit |
| c-PARP | Cell Signaling | 5625 | 1:500 | Rabbit |
| P62 | Cell Signaling | 88588 | 1:1000 | Rabbit |
| LC3B | Cell Signaling | 2775 | 1:1000 | Rabbit |
| Snail | Cell Signaling | 3879 | 1:1000 | Rabbit |
| c-Caspase3 | Abcam | Ab2302 | 1:500 | Rabbit |
| p-MLKL | Abcam | Ab187091 | 1:500 | Rabbit |
| ZEB1 | Santa Cruz | sc-515797 | 1:250 | Mouse |
| Vimentin | Santa Cruz | sc-6260 | 1:250 | Mouse |
| R-Spondin1 | R&D systems | MAB4658 | 1:500 | Mouse |
| PRELP | Invitrogen | PA5-78631 | 1:1000 | Rabbit |

**Supplementary Table 2. PCR primer sequences**

| **Gene** | **Direction** | **Primer Sequence (5’-3’)** |
| --- | --- | --- |
|  |  |  |
| h-HDAC1 | F | CTACTACGACGGGGATGTTGG |
|  | R | GAGTCATGCGGATTCGGTGAG |
| h-HDAC2 | F | ATGGCGTACAGTCAAGGAGG |
|  | R | TGCGGATTCTATGAGGCTTCA |
| h-HDAC3 | F | CCTGGCATTGACCCATAGCC |
|  | R | CTCTTGGTGAAGCCTTGCATA |
| h-HDAC4 | F | GGCCCACCGGAATCTGAAC |
|  | R | GAACTCTGGTCAAGGGAACTG |
| h-HDAC5 | F | TCTTGTCGAAGTCAAAGGAGC |
|  | R | GAGGGGAACTCTGGTCCAAAG |
| h-HDAC6 | F | AAGAAGACCTAATCGTGGGACT |
|  | R | GCTGTGAACCAACATCAGCTC |
| h-HDAC7 | F | GGCGGCCCTAGAAAGAACAG |
|  | R | CTTGGGCTTATAGCGCAGCTT |
| h-HDAC8 | F | TCGCTGGTCCCGGTTTATATC |
|  | R | TACTGGCCCGTTTGGGGAT |
| h-HDAC9 | F | AGTAGAGAGGCATCGCAGAGA |
|  | R | GGAGTGTCTTTCGTTGCTGAT |
| h-HDAC10 | F | CAGTTCGACGCCATCTACTTC |
|  | R | CAAGCCCATTTTGCACAGCTC |
| h-GAPDH | F | CCAGGTGGTCTCCTCTGACTTCAACA |
|  | R | AGGGTCTCTCTCTTCCTCTTGTGCTC |

**Legends for Supplementary Figures 1-8**

**Supplementary Figure 1. Clinical and molecular details of the 36 human derived pancreatic cancer derived organoids. A:** Number derived by biopsy versus from resection specimen, chemo-naive versus post-chemotherapy, and PurIST Classical-like versus Basal-like organoids. **B:** Results of whole exome sequencing in 29 organoids showing the tumor mutational burden (TMB), and specific gene alterations. **C:** Chromosomal analysis heat map showing the log2 of the copy number (CN) ratio (when this value is 0 it means no CN gain or loss, a single CN gain = 0.585, and a single CN loss = -1.0). **D:** No significant difference in gene losses between chemo-naive versus post-chemotherapy organoids but a significant increase in gene gains in the post-treatment organoids. **E:** A heat map of RNASeq of all of the organoids showing the PurIST classification based on the continuous score and the expression levels of the eight most common Basal-like genes (including KRT5), and the eight most common Classical-like genes (including GATA6).

**Supplementary Figure 2. Genomic and transcriptomic comparison between organoids and matched primary tissue. A:** Genomic Comparison. The oncoprint displays the most recurrent mutations observed in our cohort of 23 matched primary and organoid samples. The genes highlighted in bold are among the top mutated genes from the TCGA PDAC study. The columns in the plot represent individual samples and the mutations are color-coded if the SNV classification is a stop gain mutation or a non-synonymous mutation. KRAS mutations are observed at a frequency of 89% in most matched pairs, however, there are some exceptions where the mutation is not observed in both samples from the same patient. Tissue heterogeneity and clonal evolution are possible explanations. **B:** Transcriptomic Comparison. In the heatmap, purIST score-based classification of organoids and matched primary samples was performed. Except for a few cases, most of the organoids and matched primary tissues show similar classification results. In general, one would expect the organoids to be more transcriptionally distinct from primary tissues, due to TME influence etc.

**Supplementary Figure 3.** **Morphology of organoids from H&E and brightfield images of PDOs. A:** H&E staining of all 36 organoids. **B:** Brightfield images of organoid h33 in culture, immediately after generation from primary tissue at passage-0 and the other after passage-5, on culture days 1,3, 5, and 7**.** Blue and black scale bars: = 100 μM.

**Supplementary Figure 4. RNA expression levels of HDACs and GSK-3β, and HADC-9 levels following Metavert treatment in organoids. A:** mRNA levels are shown for ten individual HDACs 1-10, and GSK-3β relative to GAPDH in chemo-naïve organoids. h03 was Basal-like and the remaining organoids were Classical-like. **B**: mRNA levels for HDACA9 at baseline (h’n’) and following treatment with Metavert (h’n’MT). HDAC9 was the only HDAC to demonstrate mRNA HDAC following Metavert treatment.

**Supplementary Figure 5. Western blots of CD44, SOX2, and ace-H3K9, and CD44 and SOX2 mRNA levels before and after Metavert treatment.** Metavert treatment caused strong H3K9 acetylation in all eight human derived PDAC organoids, previously only shown in experimental systems^16^. `Similarly previously shown in experimental systems Metavert treatment of human organoids also strongly downregulated protein levels of the cancer stem cell markers CD44 and SOX2 associated with EMT and chemotherapy resistance^16^.

**Supplementary Figure 6. Induction of autophagy and apoptosis contributes to the anticancer activity of Metavert. A:** Apoptosis-like morphology was observed in hPDOs following treatment with Metavert (20 μM) for 72 hours, representative images for h02 are shown. **B-E:** Western blot analysis of the corresponding cell death proteins in BxPC3 and PANC1 cells after 24-72 hours of treatment with Metavert (0,5,20 μM) versus the control group (n=3; *p=0.05). **F-H:** Multiplexed IF images of representative BxPC3 cells after Metavert treatment (0, 20 μM) and positive controls (irinotecan 10 μM; TNF-α 10 μM) stained with c-PARP (green), cleaved-Caspase 3 (red), p-MLKL (yellow) and DAPI (blue). Scale bars indicated in the figures are 20 µm. **I:** BxPC3 cells were transfected with the mCherry-GFP-LC3 plasmid. After 24 hours, cells were treated with Metavert (0,5,20 μM) for another 72 hours. Chloroquine (CQ) (20 μM) which inhibits autophagy by impairing autophagosome fusion with lysosomes used as a control. **J:** BxPC3 and PANC1 and organoid h63 and h67 were treated with Metavert (0,5,20 μM) for 72 hours in the presence or absence of 24 hours of CQ pre-treatment. The expression levels of LC3-II and p62 are shown. **K:** BxPC3 and PANC1 cells were treated with Metavert in the absence or presence of cell death inhibitors necrostatin-1 (targets RIP1), ZVAD-FMK (pan-caspase inhibitor), CQ, and 3-Methyladenine (3-MA, inhibits blocks autophagosome formation via class III PI3K complex inhibition) for 24 hours. Cell viability was assayed by MTT (n=3, *p < 0.05). Scale bars indicated in the figures are 20 µm.

**Supplementary Figure 7. Metavert synergistically increased apoptosis and autophagy when combined with irinotecan. A-B:** Western blot of different cell death proteins (c-PARP, p-MLKL and LC3-I/II) in BxPC3 cells after 72 hours of treatment with Metavert and cytotoxics as shown, re-probed for GAPDH to confirm equal loading (n=3). Metavert+irinotecan induced higher levels of c-PARP (p=0.012) and LC3-I/II (p=0.014). **D-E:** representative IF localization images c-PARP (red), LC3-II (green), and DAPI (blue) after 72 hours treatment of Metavert, irinotecan and gemcitabine or combined (scale bar =100 µm).

**Supplementary Figure 8. Synergistic comparison based on tissue resources and Metavert response. A:** Comparison of IC50 and synergistic scores from the organoids generated from metastatic biopsies (metastatic) and primary resected (resected) tissues. **B:** Synergistic scores of the two groups of organoids regarding their Metavert response. p-value has been shown above in the figure.
